# Assessment of animal hosts of pathogenic *Leptospira* in northern Tanzania

**DOI:** 10.1371/journal.pntd.0006444

**Published:** 2018-06-07

**Authors:** Kathryn J. Allan, Jo E. B. Halliday, Mark Moseley, Ryan W. Carter, Ahmed Ahmed, Marga G. A. Goris, Rudy A. Hartskeerl, Julius Keyyu, Tito Kibona, Venance P. Maro, Michael J. Maze, Blandina T. Mmbaga, Rigobert Tarimo, John A. Crump, Sarah Cleaveland

**Affiliations:** 1 The Boyd Orr Centre for Population and Ecosystem Health, Institute of Biodiversity, Animal Health and Comparative Medicine, University of Glasgow, Glasgow, United Kingdom; 2 Institute of Biological and Environmental Science, University of Aberdeen, Aberdeen, United Kingdom; 3 WHO/FAO/OIE Collaborating Leptospirosis Reference Laboratory, Royal Tropical Institute, Amsterdam, The Netherlands; 4 Tanzania Wildlife Research Institute, Arusha, Tanzania; 5 Nelson Mandela African Institution for Science and Technology, Arusha, Tanzania; 6 Kilimanjaro Christian Medical Centre, Moshi, Tanzania; 7 Kilimanjaro Christian Medical University College, Moshi, Tanzania; 8 Centre for International Health, University of Otago, Dunedin, New Zealand; 9 Duke Global Health Institute, Duke University, Durham, North Carolina, United States of America; University of California Davis, UNITED STATES

## Abstract

Leptospirosis is a zoonotic bacterial disease that affects more than one million people worldwide each year. Human infection is acquired through direct or indirect contact with the urine of an infected animal. A wide range of animals including rodents and livestock may shed *Leptospira* bacteria and act as a source of infection for people. In the Kilimanjaro Region of northern Tanzania, leptospirosis is an important cause of acute febrile illness, yet relatively little is known about animal hosts of *Leptospira* infection in this area. The roles of rodents and ruminant livestock in the epidemiology of leptospirosis were evaluated through two linked studies. A cross-sectional study of peri-domestic rodents performed in two districts with a high reported incidence of human leptospirosis found no evidence of *Leptospira* infection among rodent species trapped in and around randomly selected households. In contrast, pathogenic *Leptospira* infection was detected in 7.08% cattle (n = 452 [5.1–9.8%]), 1.20% goats (n = 167 [0.3–4.3%]) and 1.12% sheep (n = 89 [0.1–60.0%]) sampled in local slaughterhouses. Four *Leptospira* genotypes were detected in livestock. Two distinct clades of *L*. *borgpetersenii* were identified in cattle as well as a clade of novel *sec*Y sequences that showed only 95% identity to known *Leptospira* sequences. Identical *L*. *kirschneri* sequences were obtained from qPCR-positive kidney samples from cattle, sheep and goats. These results indicate that ruminant livestock are important hosts of *Leptospira* in northern Tanzania. Infected livestock may act as a source of *Leptospira* infection for people. Additional work is needed to understand the role of livestock in the maintenance and transmission of *Leptospira* infection in this region and to examine linkages between human and livestock infections.

## Author summary

Leptospirosis is a globally important disease that is transmitted from animals to people and affects more than 1 million people worldwide each year. Leptospirosis is an important cause of febrile illness in northern Tanzania but little is known about the animal hosts of *Leptospira* infection for people in this area. This study aimed to evaluate the role of rodents and ruminant livestock (cattle, sheep and goats) in the epidemiology of *Leptospira* infection in northern Tanzania. The results of our study showed that ruminant livestock but not rodents are commonly infected with pathogenic *Leptospira* infection. Genetic typing identified four distinct types of *Leptospira* in livestock, including three types that were only identified in cattle, and one type that was identified in cattle, goats and sheep sampled in our study. These results indicate that livestock are a potential source of infection for people in Tanzania. This finding is important as a large proportion of the human population are employed in farming activities or keep ruminant livestock at home. Further work is needed to understand which *Leptospira* types are transmitted in our setting and to understand how livestock infection contributes to human disease.

## Introduction

Leptospirosis is a zoonotic disease caused by infection with a pathogenic serovar of *Leptospira* bacteria. Worldwide, leptospirosis is estimated to affect more than one million people and result in the loss of 2.9 million Disability Adjusted Life Years (DALYs) each year [1]. The greatest burden of leptospirosis occurs in tropical and sub-tropical areas, where people live in close contact with animal hosts and warm humid conditions facilitate environmental survival of the bacteria [1, 2]. The clinical presentation of leptospirosis ranges from a mild febrile illness to severe disease with secondary manifestations including renal failure, multiple organ dysfunction, and severe pulmonary haemorrhagic syndrome (SPHS) [3]. The reported median case fatality ratio is around 2% for uncomplicated leptospirosis and 12–40% in patients with more severe disease manifestations such as jaundice and renal failure [4]. Under-reporting of leptospirosis is thought to be common, particularly as human leptospirosis can be difficult to distinguish clinically from other tropical causes of fever such as malaria or dengue fever [5, 6].

Human infection with *Leptospira* occurs following direct or indirect contact with the urine of an infected mammalian host [5]. To date, more than 250 pathogenic *Leptospira* serovars belonging to 10 different *Leptospira* species have been described, which infect a wide variety of animal hosts [7, 8]. Rodents are common hosts of pathogenic *Leptospira* and are often considered as the most important source of human infection [3, 5]. However, many other animals including companion animals, production livestock species such as cattle and pigs, or wildlife can also carry the infection [9]. In settings where multiple hosts and serovars are present, determining the epidemiology of leptospirosis and identifying sources of human infection can be complex and challenging.

Acute leptospirosis is an important cause of human febrile disease in Tanzania. Hospital-based surveillance conducted in the Kilimanjaro Region of northern Tanzania demonstrated acute leptospirosis in 2–9% of febrile admissions [10, 11]. Estimates of the population-level incidence of leptospirosis in the Kilimanjaro Region vary over time with 11–18 cases per 100,000 per year in 2012–14 [11] and 75–102 cases per 100,000 per year in 2007–08 [12]. A large number of different *Leptospira* serogroups have been implicated in human disease although the most common predominant serogroups vary by year and by study [11]. Little is known about sources of infection for people in northern Tanzania. *Leptospira* bacteria have been isolated from cattle, pigs and a variety of small mammal species elsewhere in Tanzania [13]. However, the roles of these animal hosts as a source of infection for people in the Kilimanjaro Region remains unclear.

This study was performed to identify hosts of pathogenic *Leptospira* bacteria in northern Tanzania. To assess the role of rodents in the epidemiology of *Leptospira* infection, a cross-sectional survey of peri-domestic rodents was conducted in two districts with a high reported incidence of human leptospirosis. Sampling of cattle, sheep and goats was also performed in local slaughterhouses. The prevalence of *Leptospira* infection was determined by qPCR testing of kidney samples. Molecular typing of *Leptospira* bacteria was performed to characterise circulating *Leptospira* species and genotypes in animal hosts. Here, we discuss the results of these studies and their implications for our understanding of human and animal *Leptospira* infection in northern Tanzania.

## Methods

### Ethics statement

Ethical approval for the study was granted by the Tanzania Commission for Science and Technology (COSTECH 2012-471-ER-2005-141); Kilimanjaro Christian Medical Centre (KCMC) Ethics Committee (537); National Institute of Medical Research (NIMR), Tanzania (NIMR/HQ/R.8a/Vol.IX/1499); Tanzania Wildlife Research Institute (TAWIRI); University of Glasgow College of Medicine, Veterinary Medicine and Life Sciences Ethics Committee (200120020), and University of Glasgow Faculty of Veterinary Medicine Ethics and Welfare Committee (01a/13 & 02a/13). Written consent for study participation was obtained for each participating household. Rodent sampling was performed in accordance with UK and international guidelines for humane euthanasia [14, 15].

### Description of the study site

**Fig 1:**
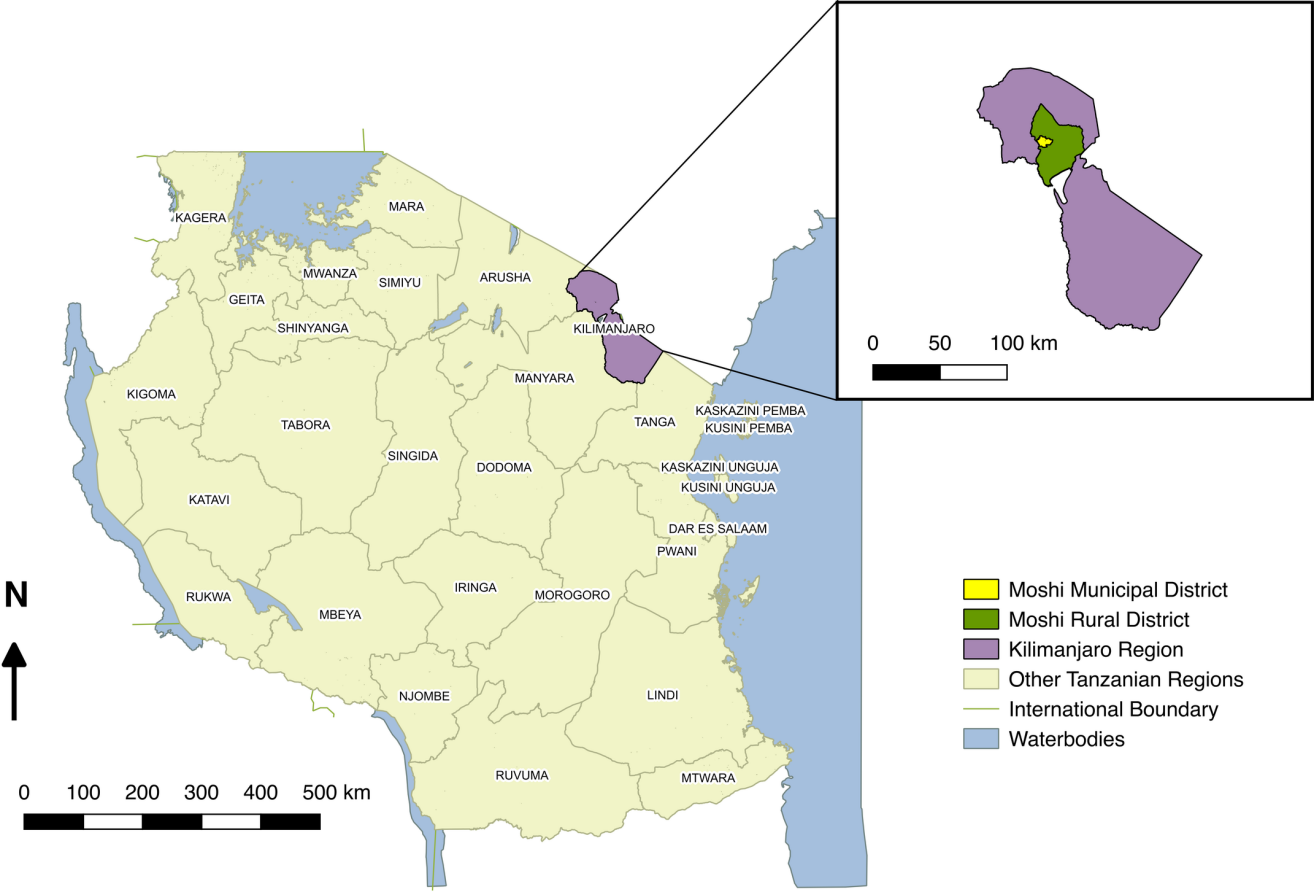
Map of Tanzania showing the administrative regions of Tanzania (main map) and the location of the Moshi Municipal and Moshi Rural Districts within the Kilimanjaro Region (inset). Maps were made using Quantum Geographic Information System (QGIS) open access software [19]. Shapefiles were obtained from Tanzania National Bureau of Statistics [20].

The study was conducted in the Kilimanjaro Region in northern Tanzania. The climate in this region follows a pattern of long rains from March to May and short rains from October to December with the coolest months coinciding with the long dry season from June to September. The region has a population of 1.64 million people, and an estimated population density of 124 people per km^2^ (national average: 51 per km^2^) [16]. The region is divided into seven districts. Two districts, Moshi Municipal and Moshi Rural (Fig 1), were chosen as the site of the study due to the high reported incidence of human leptospirosis [12] and on-going febrile disease surveillance at local hospitals (Fig 1).

Moshi Municipal District is the administrative centre of the Kilimanjaro Region. In the 2012 Tanzania National Census, the district was classified as urban and had a population of approximately 184,000 people [16]. Moshi Rural District has a population of approximately 467,000 people and is dominated by small-scale agriculture and smallholder livestock farming [16]. The environment ranges from lush high-altitude mountainous areas where coffee, bananas, and avocados dominate cash crop production, to drier low-altitude pasture land and plains where maize and beans are cultivated. Subsistence livestock farming is common. In the most recent livestock census (2008), the populations of ruminant livestock reported were 139,000 cattle and 353,000 small ruminants (sheep and goats combined) for Moshi Rural District and 2,100 cattle and 7,300 small ruminants for Moshi Municipal District (population size given to nearest 100) [17].

### Selection of study villages for cross-sectional sampling

**Fig 2:**
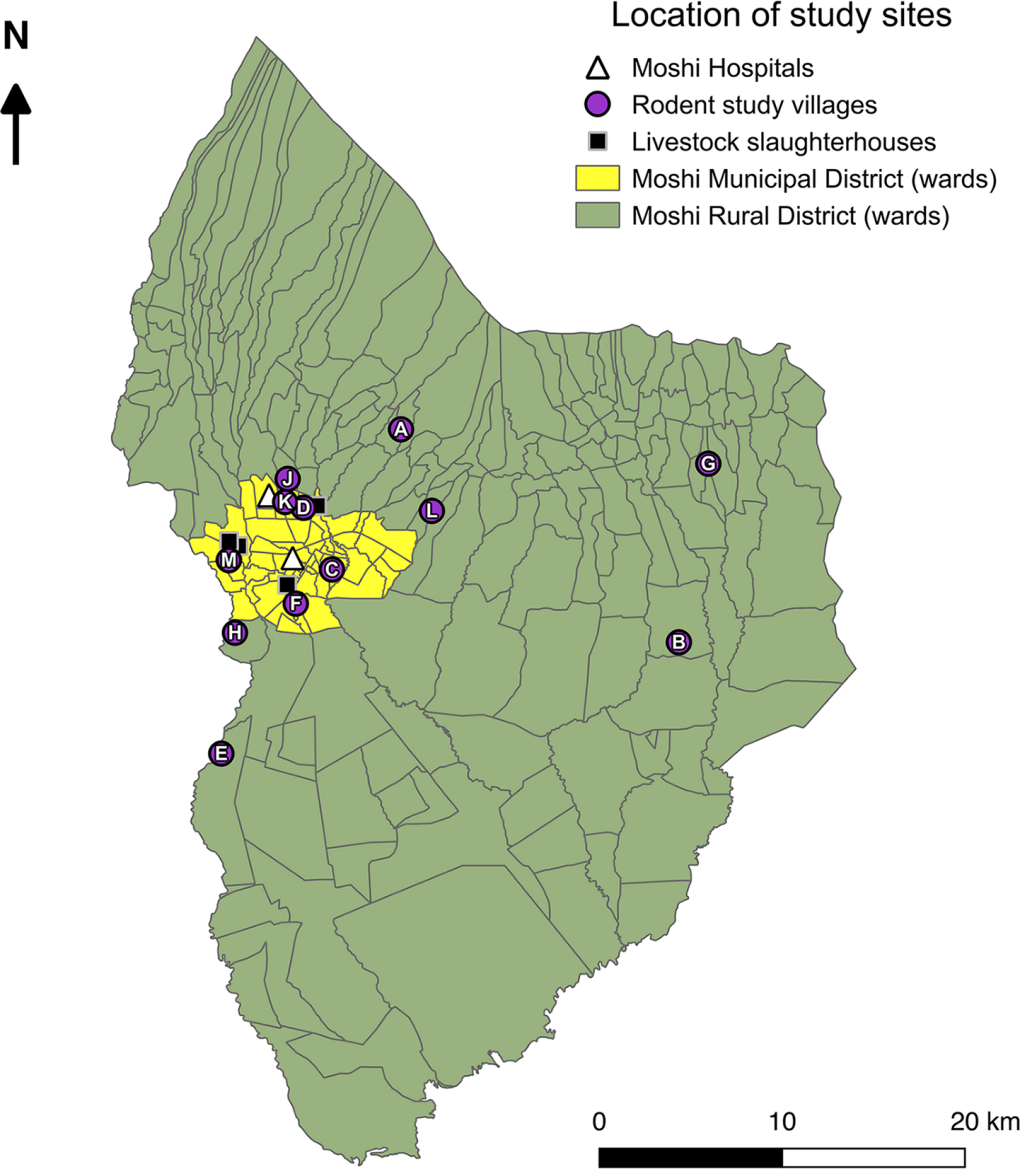
Map of Moshi Municipal and Moshi Rural Districts showing representative locations of rodent study villages and study slaughterhouses in relation to the two study hospitals (Kilimanjaro Christian Medical Centre (KCMC) and Mawenzi Regional Referral Hospital (MRRH). Maps were made using Quantum Geographic Information System (QGIS) open access software [19]. Districts shapefiles were obtained from Tanzania National Bureau of Statistics [20].

A cross-sectional survey was performed to determine the prevalence of *Leptospira* infection in peri-domestic rodents within the catchment area of two hospitals (Kilimanjaro Christian Medical Centre (KCMC) or Mawenzi Regional Referral Hospital (MRRH)) that previously identified a high prevalence of acute leptospirosis in patients with febrile illness [10, 11]. The geographical sampling frame was composed of villages within Moshi Municipal and Moshi Rural Districts from which people had sought health care and been enrolled in fever surveillance studies at KCMC and MRRH in the preceding years (2012–2014). One village was selected by convenience as a pilot village (2013) and eleven study villages were selected at random (Fig 2). Consent for study participation was obtained from the Village Chairperson of each study village, who also provided a list of sub-villages within their villages. A single sub-village was selected at random as the sampling location within each study village. The population size of selected sub-villages ranged from 916 to 4320 people (Moshi Municipal: 1039–4320 people; Moshi Rural: 916–3926 people) [18]. Using a reported average household size of 4, this equates to approximately 229 to 1080 households per sub-village (Moshi Municipal: 260–1080 households; Moshi Rural: 229–935 households) [16, 18].

### Mapping of cross-sectional study

Study maps (Figs 1 & 2) were made using Quantum Geographic Information System (QGIS) open access software [19]. Shapefiles for Tanzania country boundaries, regions and districts from the most recent census were obtained from Tanzania National Bureau of Statistics [16, 20]. A single representative location for each study village was defined by recording the GPS co-ordinates for the administrative centre of each sampled sub-village.

### Rodent trapping and sampling

Rodent trapping was performed in three sampling periods: 1) May-June 2013 (wet season); 2) May-June 2014 (wet season); and 3) August-September 2014 (dry season). The target sample size was 50 rodents per sub-village to give sufficient power (α = 0.95, β = 0.8) to detect a minimum *Leptospira* infection prevalence of 10% [21–24]. Based on a predicted average trap success of 12.5% [22, 25], 100 traps were set for a target of four nights to give a trapping effort of 400 trap nights per sub-village with the exception of the pilot village (A), where only 50 traps were used. Following initial trapping (villages A & B), the number of nights was increased to an average of eight (trapping effort of 800 trap nights) per sub-village due to lower than expected trapping success.

Sampling transects were established in each sub-village using a method based on the World Health Organization (WHO) Expanded Program for Immunization (EPI) random walk method for cluster sampling [26, 27]. The administrative centre of each sub-village was used as the starting point for sampling transects. The direction of each transect was determined at random within the sub-village (defined by spinning a pen in the field) and ran from the centre of the sub-village to its peripheral boundary. Households were recruited along each transect ensuring a minimum distance of 50 metres between each household until 20 households had been recruited.

Five rodent traps were set in each participating household. In 2013, four large Sherman traps (HB Sherman Traps, Tallahassee, USA. Dimensions: 7.6 x 8.9 x 22.9 cm) and one small Sherman trap (dimensions: 5.1 x 6.4 x 22.9 cm) were set in each household. In 2014, the trapping approach was adjusted and one large Sherman trap per household was replaced with a Tomahawk trap (Tomahawk Live Trap, Hazelhurst, USA. Model 602; dimensions 12.7 x 12.7 x 40.6 cm). Traps were placed in kitchens, food storage areas, and animal housing areas within each household and in sheltered outdoor areas within each compound (e.g. adjacent to animal houses, fence lines and in log piles). A stiff mixture of peanut butter and oats and chopped carrots was used to bait Sherman traps. Dried fish was used to bait Tomahawk traps. Traps were checked and reset each morning. Traps containing rodents were removed and replaced. Trapped rodents were euthanised by terminal halothane anaesthesia and cervical dislocation. The species of each trapped rodent was determined by observation of phenotypic characteristics and measurement of morphometric features [28, 29]. Rodent sex and age class (mature or immature) was determined based on external sexual characteristics [29]. A full necropsy and tissue sampling was performed. For detection of *Leptospira* infection, one kidney from each rodent was collected and preserved in 70–96% ethanol at room temperature prior to testing by real-time PCR (qPCR).

For a subset of rodents, kidney tissue was also collected for *Leptospira* culture. Culture was attempted opportunistically during the randomised cross-sectional survey in Villages C, D, E & M based on availability of culture media. In addition, to maximise the chance of *Leptospira* culture success, the village with the highest trap success in the cross-sectional survey (Village F) was re-visited in September 2014 for repeat rodent trapping and sampling for culture. In this village, trapping was repeated in the 20 previously recruited households using the same strategy (100 traps x 8 nights). Rodent sampling was performed as described above, and kidney tissue was collected for qPCR and culture.

### Slaughterhouse sampling of ruminant livestock

Ruminant livestock (cattle, goats and sheep) was sampled in slaughterhouses within the Moshi Municipal District. Five slaughterhouses were selected for livestock sampling in liaison with the District Veterinary Officer based on high slaughter throughput (ranging from 14 and 210 cattle per week), accessibility of location and cooperation from livestock field officers responsible for meat hygiene inspection at each of the slaughterhouses. GPS co-ordinates were recorded at each participating slaughterhouse (Fig 2). The target sample size for cattle (n = 323) was selected to give the study sufficient power to estimate the prevalence of infection with a precision of 5% based on seroprevalence estimates of 30% [30]. Goat and sheep sampling was performed opportunistically at the same slaughterhouses.

Livestock sampling was performed between May 2013 and September 2014. A maximum of ten animals per species were sampled per slaughterhouse per day. The source (region, district and market of origin), approximate age (adult vs. juvenile), gender, and breed (indigenous, exotic or cross-breed) were recorded for each animal. Kidney samples were collected during evisceration into a clean, labelled, single-use Ziplok bag. Following surface sterilisation with a flamed blade, samples of kidney tissue (approximately 3 x 1 x 1 cm) spanning the cortico-medullary junction were taken using a sterile blade and placed directly into 70–96% ethanol prior to testing by qPCR. Samples of kidney tissue were also collected for *Leptospira* culture from an opportunistically selected subset of cattle and goats.

### DNA extraction and qPCR testing for Leptospira infection

The prevalence of renal *Leptospira* infection in livestock and rodents was determined by qPCR testing. DNA was extracted from 25 milligrams (mg) of kidney tissue preserved in ethanol using the QIAamp DNA Mini Kit spin-column protocol for DNA purification from tissues (Qiagen, Maryland, USA). The DNA concentration was quantified using a NanoDrop spectrophotometer (ThermoScientific, Waltham, MA) and stored at -20°C prior to qPCR testing. DNA extracts were tested for pathogenic *Leptospira* spp. using a *lip*L32 TaqMan qPCR assay run on the ABI 7500 Real-Time PCR system (Applied Biosystems, Foster City, CA) as previously described [31, 32]. Amplification of a 245 bp product was performed using the primer set: lipL32-45F (5’-AAG CAT TAC CGC TTG TGG TG-3’) and lipL32-286R (5’-GAA CTC CCA TTT CAG CGA TT-3’), and a 19-bp 5’FAM-labelled probe with a 3’BHQ quencher dye (FAM-5’-AA AGC CAG GAC AAG CGC CG-‘3-BHQ1). Low concentration ROX (50nmol/L) was added to the final reaction mix as a passive reference to improve the diagnostic sensitivity and specificity of the assay [33]. DNA extracts were diluted 1:10 in PCR grade water to reduce the effects of PCR inhibitors. Amplifications were performed using 5μl of diluted template DNA (approximately 50 to 150ng) per 25μl qPCR reaction. Samples were tested in duplicate. Two replicates of a *Leptospira* positive control, *L*. *interrogans* serovar Copenhageni Strain Wijnberg were also run per reaction plate. Control DNA was sourced from the WHO/FAO/OIE Collaborating Leptospirosis Reference Laboratory in Amsterdam and tested at a concentration of 1 pg of DNA (approximately equal to 10^2^ genomic equivalents) per 25μl qPCR reaction. In addition, two replicates of a non-template extraction control, and two replicates of PCR-grade water were included on each test plate. Reaction profiles were analysed using Applied Biosystems 7500 System Sequence Detection (SDS) Software Version 1.2.4 (Applied Biosystems, Carlsbad, CA 2001–2004). A qPCR plate run was considered valid when all negative controls were negative and at least one replicate of the *Leptospira* positive controls amplified with cycle threshold (Ct) value < 40. Samples were considered positive when at least one test well amplified the *lip*L32 target with a Ct value < 40.

### Typing of Leptospira from qPCR-positive samples

For qPCR-positive samples, the infecting *Leptospira* species was determined through amplification and sequencing of a conserved 470-bp segment of the *sec*Y gene previously shown to have phylogenetic discriminatory power for pathogenic *Leptospira* species [34, 35]. PCR assays optimized for use in eastern Africa were run at the University of Aberdeen following published protocols [36]. Amplifications were performed using 5μl undiluted template DNA in a 25μl PCR reaction using the primer set: secYFd (5’-ATG CCG ATC ATY TTY GCT TC-3’) and secYR3 (5’-TTC ATG AAG CCT TCA TAA TTT CTC A-3’). All PCR assays included one non-template control (PCR grade water) per five test samples and a positive control of DNA extracted from a pure isolate of *L*. *interrogans* or *L*. *borgpetersenii*. PCR products were visualised by gel electrophoresis on a 1.5% agarose gel and purified using the QIAquick PCR Purification Kit following manufacturer’s instructions (Qiagen, Maryland, USA). Purified products were quantified using a Nanodrop ND1000 spectrophotometer (ThermoScientific, Massachusetts, USA) and sequenced by Eurofins Genomics GmbH (Ebersburg, Germany).

### Leptospira culture

*Leptospira* culture was performed from kidney tissue samples collected from a total of 98 rodents, 100 cattle, and 49 goats. Following kidney collection, the renal capsule was sterilised using a hot flamed blade and approximately 25 mg of kidney tissue was dissected across the cortico-medullary junction. Tissue was immediately homogenised in 1ml of Ellinghausen-McCullough-Johnson-Harris (EMJH) culture media supplemented with 0.4mg/ml of fluorouracil (5’FU) (EMJH-5FU media) supplied by the WHO/FAO/OIE Collaborating Leptospirosis Reference Laboratory in Amsterdam. A ten-fold dilution series (1:10, 1:100, 1:1000) was prepared in three tubes with 5 ml of EMJH-5FU. Inoculated aliquots of culture media were shipped to the WHO/FAO/OIE Collaborating Leptospirosis Reference Laboratory in Amsterdam for *Leptospira* isolation. Cultures were incubated at 30°C and checked for *Leptospira* growth by dark-field microscopy every four weeks for three months and then again after six months of incubation. Positive cultures were confirmed by *sec*Y qPCR [37] and sub-cultured in EMJH media prior to typing.

### Typing of Leptospira isolates

*Leptospira* isolated by culture were typed using serological and genetic methods at the WHO/FAO/OIE Collaborating Leptospirosis Reference Laboratory in Amsterdam. Serological typing of pathogenic *Leptospira* isolates was performed by microscopic agglutination test in two stages. First, a panel of polyclonal rabbit antisera raised against 24 *Leptospira* serogroups was used to determine the serogroup of isolates [38]. Subsequently, a panel of 18 serovar-specific mouse monoclonal antibodies was used to determine the isolate serovar [39, 40]. Sequence type was determined using a multi-locus sequence typing (MLST) scheme targeting seven *Leptospira* housekeeping genes (*glmU*, *pntA*, *sucA*, *tpiA*, *pfkB*, *mreA* and *caiB)* following published protocols [41]. PCR amplicons were sequenced by Macrogen Europe (Amsterdam, Netherlands). Trimmed sequences were aligned against reference sequences for the MLST scheme (obtained from PubMLST; *Leptospira* Scheme #1: http://pubmlst.org/leptospira/) to generate a unique allelic profile for each isolate [42, 43]. Finally, each allelic profile was compared to an online database of 223 profiles to determine the sequence type (ST) and *Leptospira* serovar [41].

### Phylogenetic analysis

Phylogenetic analysis was performed using MEGA7.0 software [44]. *Leptospira sec*Y sequences from qPCR positive samples and *Leptospira* isolates obtained in this study were trimmed and then aligned using the ClustalW algorithm in MEGA with *sec*Y sequences from 128 *Leptospira* reference serovars obtained through GenBank [34, 45]. The model test function in MEGA was used to select the most appropriate nucleotide substution model for the aligned sequences. Phylogenetic analysis was performed using a maximum likelihood method with 500 bootstrap repeats to generate the final phylogenetic tree.

### Statistical analysis

Adjusted trap success was used as a measure of relative rodent abundance in each sub-village [46]. Adjusted trap success was calculating by dividing the total number (n) of rodents caught per sub-village by the corrected number of trap nights (Total number of trap nights (number of traps x number of nights) minus lost trap nights (sum of number of closed, damaged or lost traps / 2) and expressed as a percentage). Statistical analysis was performed in R [47]. Two-sample T-tests were used to compare the adjusted trap success and proportion of households with rodents between the two study districts. Binomial confidence intervals for point prevalence estimates (Wilson method) were calculated using the Hmisc package [48]. Fisher’s exact tests were performed to compare the prevalence of infection between animal species, and between sex and age groups within-species.

## Results

### Cross-sectional surveillance of peri-domestic rodents

Overall, five villages in Moshi Municipal District and seven villages in Moshi Rural District were selected for inclusion in this study. A summary of selected village details is given in Table 1. During the randomised cross-sectional survey, 351 rodents were trapped across the 12 selected villages. Rodents were trapped in 60.0% of all participating households. The adjusted trap success by village ranged from 1.94 to 10.4% (median = 4.42%). Overall, no significant differences were observed in the adjusted trap successes (two sample t-test: p = 0.690) or average proportion of households with trapped rodents (two-sample t-test: p = 0.124) between the two study districts. In addition, a further 33 rodents (*R*. *rattus*: n = 21, 63.6% and *M*. *musculus*: n = 12, 36.4%) were trapped from 80.0% of households during repeat sampling in village F (adjusted trap success of 4.42%).

**Table 1:** Summary of rodent trapping effort and success by village.

Village ID	A (Pilot)	B	C	D	E	F	F_2_ ‡	G	H	J	K	L	M	Total
District	Moshi Rural	Moshi Rural	Moshi Municipal	Moshi Municipal	Moshi Rural	Moshi Municipal	Moshi Municipal	Moshi Rural	Moshi Rural	Moshi Rural	Moshi Municipal	Moshi Rural	Moshi Municipal	-
Season and year	Wet 2013	Wet 2013	Wet 2013	Wet 2013	Wet 2013	Wet 2014	Dry 2014	Wet 2014	Wet 2014	Wet 2014	Dry 2014	Dry 2014	Dry 2014	-
**Sampling nights per village** (n)	3	4**	7	10	8	8	8	8	8	8	8	8	8	**88**
**Adjusted trap nights per village** (n)	143*	304	650	932	738	731	747	773	748	742	722	751	751	**7985**
**Rodents trapped** (n)	14	13	31	25	39	76	33	15	35	20	23	22	38	**351**
**Adjusted trap success** (%)	9.79	4.28	4.77	2.68	5.28	10.8	4.42	1.94	4.69	2.70	3.19	2.93	5.06	**4.42%**
**Households with trapped rodents**	60.0%	45.0%	60.0%	50.0%	50.0%	90.0%	80.0%	40.0%	60.0%	65.0%	55.0%	60.0%	65.0%	**60.0%**

*For pilot village sampling, 5 traps were placed in 10 households for a total of 3 sampling nights.

**For the first night in Village B, traps were set at only 10 households. A further 10 households were recruited the following day.

‡Repeat sampling was conducted in village F (shown as F2) at the end of the study period to increase the chance of *Leptospira* culture success.

In total, 384 rodents were trapped in this study and were tested for *Leptospira* infection. Of these, 221 (57.6%) were female and 225 (58.6%) were classified as sexually mature based on external sexual characteristics. The most common species trapped was the black rat (*Rattus rattus*) (n = 320, 85.1%). Other species included house mice (*Mus musculus*: n = 44, 11.5%); multimammate mice (*Mastomys natalensis*: n = 8, 2.08%); spiny mice (*Acomys* spp.: n = 6, 1.56%); African pygmy mice (*Mus minutoides*: n = 3, 0.781%); and striped bush squirrels (*Paraxerus flavovittis*: n = 3, 0.781%).

### Slaughterhouse sampling of livestock

Kidney samples were collected from 452 cattle, 167 goats, and 89 sheep. Cattle were sampled at all five slaughterhouses included in this study (median per site = 70; range = 6–273). Opportunistic sampling of sheep was performed at three slaughterhouses (median = 40; range = 2–47) and goats at two slaughterhouses (range = 12–141, slaughterhouse information not recorded for 14 animals). Based on visual assessment of physical characteristics, 439 (97.1%) cattle, 165 (98.8%) goats and 88 (98.9%) sheep were classified as indigenous breeds. The majority of animals were male (cattle: n = 370, 81.9%; goats: n = 117, 70.1%; and sheep: n = 47, 53.8% of sheep) and 93.2% of animals were adult (cattle: n = 405, 89.6%; goats: n = 135, 80.8%; and sheep: n = 77, 86.5%).

Almost all ruminant livestock sampled in this study originated from areas outside the core study districts of Moshi Municipal and Moshi Rural (S1 Table). Of 452 cattle sampled, 381 (84.3%) originated from the Manyara Region (Fig 1), mainly from the districts of Mbulu (n = 296) and Babati (n = 65). Of five cattle that originated from the Kilimanjaro Region, only one originated from either of the Moshi districts (Moshi Rural District, n = 1). All small ruminants sampled in this study originated from either the Arusha or Manyara Regions (S1 Table).

### Leptospira qPCR results

Renal infection with pathogenic *Leptospira* spp. was detected by *lip*L32 qPCR in 32 (7.1%) cattle, 2 (1.2%) goats, and 1 (1.1%) sheep (Table 2). *Leptospira* infection was not detected in any of 384 rodent kidney samples tested by *lip*L32 qPCR (Table 2). Statistically significant differences in the prevalence of infection were detected in pairwise comparisons between cattle and small ruminants, and cattle and rodents (Fisher’s Exact Test, p < 0.05). The odds ratio (OR) of cattle *Leptospira* infection was 6.26 (95% confidence interval (CI): 1.57–54.5) when compared to goat infection; and 6.75 (95% CI: 1.10–278) when compared to sheep infection. Compared to rodents, cattle were also significantly more likely to be infected with *Leptospira* (95% CI: 7.41 –Inf). No significant differences in infection prevalence were observed in pairwise comparisons between goats, sheep or rodents (Fisher’s Exact Test, p > 0.05). For ruminant livestock species, no significant differences were observed in infection prevalence by qPCR between male and female, or adult or juvenile animals (Fisher’s exact tests; p > 0.05).

**Table 2:** Results of *Leptospira lip*L32 qPCR testing of kidneys from peri-domestic rodents and ruminant livestock (cattle, goats and sheep).

Animal host	Number tested by *lip*L32 qPCR	*Leptospira* prevalence [Binomial 95% confidence interval]
**Rodents**	384	0.00% [0.0–0.99%]
**Cattle**	452	7.08% [5.06–9.82%]
**Goats**	167	1.20% [0.33–4.26%]
**Sheep**	89	1.12% [0.06–6.09%]

### Leptospira culture results and isolate typing

*Leptospira* was successfully isolated from four cattle kidneys from the subset of cattle tested by *Leptospira* culture (n = 100). All four *Leptospira* isolates derived from cattle kidneys were typed as *L*. *borgpetersenii* serovar Hardjo (Hardjo-bovis), serogroup Sejroe (ST 152) [43]. No *Leptospira* growth was detected from the subset of rodents (n = 98) or goat samples (n = 49) that were tested for *Leptospira* infection by culture.

### Phylogenetic analysis from qPCR-positive samples

**Fig 3:**
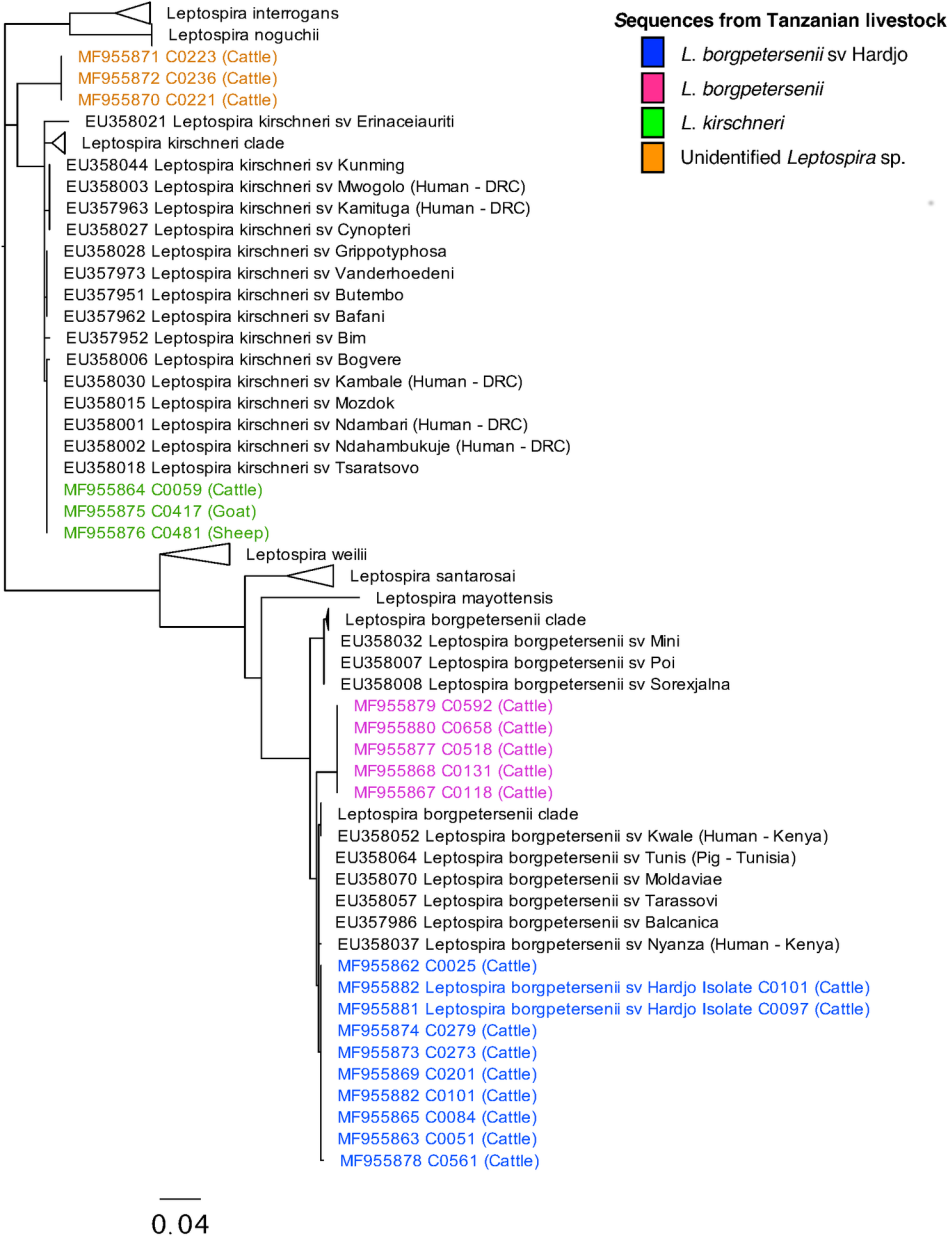
Phylogenetic tree showing the relatedness of the *Leptospira sec*Y gene (434-bp fragment) derived from qPCR-positive livestock samples. The phylogenetic tree was constructed using the maximum likelihood method based on the Tamura-Nei nucleotide substitution model [73]. The tree with the highest log likelihood is shown and drawn to scale with branch lengths measured in the number of substitutions per site. Sequences from this study are labelled with unique identifiers (C0025-C0658); host species; and GenBank accession numbers (MF955862 to MF955882). Sequence from reference *Leptospira* serovars are also shown [34]. Expanded clades show reference serovars closely related to study genotypes. More distantly related species clades are collapsed and shown with species labels only. Host and country locations shown for Africa isolates are show in parentheses. Sequences from this study that show 100% identity with *L*. *borgpetersenii* serovar Hardjo are highlighted in blue; non-Hardjo *L*. *borgpetersenii* sequences are highlighted in pink; *L*. *kirschneri* sequences are highlighted in green and sequences without an attributed species are highlighted in orange. Abbreviations: (sv) serovar; DRC (Democratic Republic of Congo).

Identification of infecting *Leptospira* species by amplification and sequencing of the *sec*Y gene was successful for 19 (54.3%) of 35 qPCR-positive kidney samples (Table 3). *L*. *borgpetersenii* was the most common infecting *Leptospira* species and was identified in 13 (72.2%) of 17 cattle samples with *sec*Y sequence available for analysis. Phylogenetic analysis revealed two distinct clades of *L*. *borgpetersenii* sequence (Fig 3). Sequences from eight cattle samples showed 100% sequence identity with *L*. *borgpetersenii* serovar Hardjo isolates obtained in this study (Fig 3: Isolate C0097 and C0101). Sequences from five cattle samples formed a separate clade within the *L*. *borgpetersenii* species, which was distinct from all reference sequences.

**Table 3:** Infecting *Leptospira* species based on *sec*Y sequencing from qPCR positive samples from cattle, goats and sheep.

*Leptospira* species	Cattle	Goats	Sheep
*Leptospira borgpetersenii*	13	0	0
*Leptospira kirschneri*	1	1	1
Unidentified *Leptospira* species	3	0	0
*sec*Y sequence not available	15	1	0
**Total qPCR positive samples**	**32**	**2**	**1**

*Leptospira kirschneri*, was identified in qPCR-positive samples from one cattle, one goat, and one sheep. Sequences from small ruminants (Fig 3: C0417 and C0481) and one bovine (Fig 3: C0059) showed 100% identity to each other as well as to several reference serovars including three serovars isolated human leptospirosis cases in the Democratic Republic of Congo (DRC: Kambale (EU358030), Ndambari (EU358001) and Ndahambukuje (EU358002)).

Infecting *Leptospira* species could not be determined by *sec*Y sequence analysis for a clade of three cattle samples (Fig 3: C0221, C0223 and C0236). In the final phylogenetic tree, the clade containing these sequences appeared most closely related to *L*. *kirschneri* but showed only 95% similarity with the closest available reference sequences. GenBank searches also failed to identify any more similar *Leptospira* species or serovars.

## Discussion

In this investigation of animal hosts of pathogenic *Leptospira* in northern Tanzania, *Leptospira* infection was detected in ruminant livestock but not in rodents sampled in two districts with a high reported incidence of human leptospirosis [10, 11]. No evidence of infection was detected in any of 384 peri-domestic rodents trapped in a cross-sectional survey conducted across a two-year period at 12 randomly selected sites. In contrast, slaughterhouse sampling of ruminant livestock detected *Leptospira* infection in cattle (7.06%), goats (1.20%) and sheep (1.11%). Two infecting *Leptospira* species were detected in ruminant livestock, including *L*. *borgpetersenii* in cattle and *L*. *kirschneri* in cattle, goats and sheep. A novel *Leptospira* genotype was also detected in cattle that showed relatively little sequence similarity (95%) to known *Leptospira* species.

The absence of *Leptospira* infection in the rodents is a notable finding of this study. Worldwide, rodents are frequent carriers of pathogenic *Leptospira* bacteria [3, 6] and are often described as the most common source of human infection [3]. However, the lack of detectable infection in our study, which was conducted in two districts where the incidence of human leptospirosis is known to be high [10, 11], indicates that peri-domestic rodents are not a major source of *Leptospira* infection for people in this area. Although these results were unexpected, we consider that they are robust. Diagnostic protocols used to test rodent samples were consistent with those used in other species (e.g. cattle) that yielded positive results. Rodent sampling was performed at 12 randomly selected villages over a two-year period and the total sample size achieved by our study (n = 384) had sufficient statistical power to demonstrate freedom from infection at the 95% confidence level, even allowing for a low prevalence of infection (e.g. 1.0%) [21, 49].

The reasons for a lack of detectable *Leptospira* infection in the rodents sampled in our study are unclear. *Rattus rattus*, the most common species sampled in our study, is globally widespread invasive rodent species that has been demonstrated as a carrier host of *Leptospira* infection in other settings [23, 50, 51]. Infection has been reported in these species in other African countries [52], including in a study conducted by the authors (KJA, JEBH, AA, RAH) in neighbouring Kenya, where *Leptospira* was detected in *R*. *rattus* (9.1%; n = 33) in the Kibera slums [22]. However, to date, no published studies of *R*. *rattus* in Tanzania (e.g. [13, 24, 53]) have demonstrated *Leptospira* infection by culture or PCR. Therefore, despite their prominent role in other settings, there is very little evidence to suggest that this species are important hosts of *Leptospira* in northern Tanzania.

To date, *Leptospira* infection has only been reported in indigenous rodent species such as the African pouched rats (*Cricetomys* spp.) and multimammate mice (*Mastomys natalensis*) [13, 54] that typically live outside of domestic environments. Although both rodent species are reported to live in the Kilimanjaro Region [28], *Cricetomys* was not trapped in our study and *M*. *natalensis* was trapped in very low numbers (n = 8) that may have been insufficient to detect low levels of infection in this host population. Another notable absence in the study was the lack of Norway rats (*Rattus norvegicus*), which is considered the definitive maintenance host of several *Leptospira* serovars including *L*. *interrogans* serovars Copenhagenii and Icterohaemorrhagiae worldwide [9, 55]. The apparent absence of key maintenance hosts of rodent-associated *Leptospira* serovars such as *Cricetomys* or *R*. *norvegicus* at our study sites is one possible explanation for the lack of infection in the rodents trapped and tested in this study.

In contrast, cattle *Leptospira* infection appears to be widespread across Tanzania. In this study, bovine *Leptospira* infection was detected in cattle originating from Manyara, Arusha, Dodoma, Singida and Tanga Regions (S1 Table). Infection has also been reported in cattle sampled in the Morogoro Region [56]. A degree of caution should be exercised in extrapolating estimates of cattle *Leptospira* prevalence from slaughterhouse studies to the source population. Selection biases for animals sent for slaughter and the potential for increased probability of infection associated with mixing of animals in markets and during transport may increase the prevalence of some infections in slaughterhouse populations [57, 58]. Further sampling of resident livestock in the study districts is necessary to understand the local prevalence and epidemiology of infection in these populations.

Demonstration of renal *Leptospira* carriage in small ruminant hosts in this study is a novel finding for sub-Saharan Africa. *Leptospira* infection is well-documented in small ruminants in other parts of the world (e.g. goats in Brazil [59] and sheep in New Zealand [60]) but there have been few studies of small ruminants as hosts of *Leptospira* infection in the African continent. Goats and sheep are important production livestock in Tanzania [61]. Small ruminant ownership is common and people live in close contact with their livestock in our study area [62]. Detection of renal infection in goats and sheep demonstrates that small ruminants in this setting also carry and shed pathogenic *Leptospira* in this setting and corroborates serological findings from elsewhere in Tanzania [63]. Small ruminants therefore also have the potential to act as sources of infection for people.

Multiple species and genotypes of pathogenic *Leptospira* were detected in infected ruminant livestock sampled in this study. *Leptospira borgpetersenii* was the predominant species infecting cattle. *L*. *borgpetersenii* serovar Hardjo was isolated from four cattle, supporting previous serological evidence for the presence of this serovar in Tanzania [63–66]. *L*. *borgpetersenii* sequence was also detected in 13 (76.5%) of 17 qPCR cattle with successful *sec*Y amplification. Sequences derived from eight qPCR-positive cattle samples were identical to those from *L*. *borgpetersenii* serovar Hardjo isolates. A second *L*. *borgpetersenii* genotype was detected in 5 (29.4%) cattle samples, which showed only 98% identity to the most similar reference serovars. GenBank BLAST searches identified *Leptospira* qPCR-positive samples with identical *sec*Y sequences in cattle from Brazil (KP862647.1) [67]. The presence of this *L*. *borgpetersenii* type in multiple international cattle populations suggests that this *Leptospira* type could be globally widespread in cattle.

*Leptospira kirschneri* was the second *Leptospira* species identified in ruminant livestock species. *L*. *kirschneri* sequences derived from cattle, goats and sheep in this study showed 100% identity to each other and to seven other reference serovars (serovars Bim, Bogvere, Kambale, Mozdok, Ndambari, Ndahambukujue, Tsaratsovo). Two serovars, *L*. *kirschneri* serovar Grippotyphosa and *L*. *kirschneri* serovar Sokoine, have previously been isolated from Tanzanian cattle and showed a high degree of similarity to *L*. *kirschneri* genotypes detected in this study (> 99%) [13, 56]. Notably, a clade of novel *sec*Y sequences was also detected in cattle qPCR-positive samples that could not be attributed to any *Leptospira* species by phylogenetic analysis. Sequences derived from three cattle infections were identical to each other but distinct from any reference sequences used in the phylogenetic analysis for this study. BLAST searches conducted in GenBank also failed to identify any similar sequences from other studies. Two possible explanations exist to describe the relationship of this clade of novel sequences to the rest of the *Leptospira* genus. First, these sequences could represent a divergent clade of *L*. *kirschneri*, which is the most similar known *Leptospira* species. However, sequence variation of 5% in the *sec*Y gene is the reported threshold of the difference observed between *Leptospira* species [34]. Therefore, an alternative explanation is that this clade represents a new and previously undescribed *Leptospira* species. Further work is needed to determine the species and fully characterise this novel *Leptospira* genotype.

The *sec*Y single-locus genotyping approach is this study provides a robust initial assessment of the diversity of *Leptospira* species circulating in Tanzanian livestock. The high degree of similarity between some of the livestock sequences identified in this study and sequences from human infections elsewhere in sub-Saharan Africa (e.g. DRC and Kenya, see Fig 3) suggests that livestock may be an important source of *Leptospira* infection for people across the eastern and central African region. To date, there are no *sec*Y sequences derived from human *Leptospira* infection in northern Tanzania, limiting our ability to use genomic data to compare infecting *Leptospira* species between human and livestock populations. Serological data from human cases in Tanzania does exists [10, 11] but the poor correlation between genotype and serogroup for *Leptospira* bacteria limits our ability to robustly link these data to attribute sources of *Leptospira* infection [7, 68]. However, epidemiological studies have identified milking cattle, feeding and cleaning cattle and handling cattle waste as significant risk factors for human *Leptospira* infection in Moshi and neighbouring regions [69, 70]. These findings suggest that cattle are indeed an important source of *Leptospira* infection for people in northern Tanzania and provide a strong rationale for further investigation linked human and cattle populations to better understand the relationship between human and bovine infection.

Overall, our study makes a substantial contribution to the growing body of evidence that livestock play an important role in the epidemiology of human leptospirosis in sub-Saharan Africa. Although the contribution of other species cannot be ruled out, contact with livestock has been demonstrated as an important risk factor for human *Leptospira* infection in northern Tanzania [70]. Occupational exposure to infected livestock is known to be an important risk factor for human leptospirosis in other settings [71] and currently more than 75% of the Tanzanian population is estimated to be employed in the agriculture sector [61]. Given the importance of leptospirosis as a cause of human febrile illness in Tanzania [72], quantifying the contribution of livestock-associated leptospirosis to human health and understanding the factors that support the maintenance and transmission of pathogenic *Leptospira* in livestock populations are important priorities for future leptospirosis and public health research.

## Supporting information

S1 Table Districts of origin for ruminant livestock sampled in this study. (DOCX)
