# Supplementary material for: Assessment of animal hosts of pathogenic Leptospira in northern Tanzania
Source: PLoS Negl Trop Dis. 2018 Jun 7;12(6):e0006444. doi: 10.1371/journal.pntd.0006444 (PMC5991636; doi:10.1371/journal.pntd.0006444)
Supplement: S1 Table — (DOCX) [file pntd.0006444.s001.docx]

**Supplementary Material Table S1: Districts of origin for ruminant livestock sampled in this study**

| **Region** | **District** | **Cattle sampled (n)** | **Number of positive cattle (%)** | **Goats sampled (n)** | **Number of positive goats (%)** | **Sheep sampled (n)** | **Number of positive sheep (%)** |
| --- | --- | --- | --- | --- | --- | --- | --- |
| Arusha | Arumeru | 9 | 0 | - | - | - | - |
|  | Longido | 4 | 0 | - | - | - | - |
|  | Meserani | 1 | 0 | - | - | - | - |
|  | Minjingu | 1 | 0 | - | - | - | - |
|  | NA | - | - | 52 | 0 | 2 | 0 |
| Dodoma | Kondoa | 6 | 0 | - | - | - | - |
|  | NA | 6 | 1 (16.7%) | - | - | - | - |
| Kilimanjaro | Moshi Rural | 1 | 0 | - | - | - | - |
|  | Mwanga | 4 | 0 | - | - | - | - |
| Manyara | Babati | 65 | 4 (6.15%) | - | - | 32 | 1 (3.13%) |
|  | Mbulu | 296 | 22 (7.43%) | - | - | - | - |
|  | Simanjiro | 6 | 2 (33.3%) | - | - | - | - |
|  | NA | 20 | 1 (5.00%) | 115 | 2 (1.74%) | 47 | 0 |
| Mwanza | Kwimba | 4 | 0 | - | - | - | - |
| Singida | Manyoni | 18 | 1 (5.56%) | - | - | - | - |
| Tabora | Igunga | 6 | 0 | - | - | - | - |
| Tanga | Handeni | 1 | 1 (100%) | - | - | - | - |
|  | Korogwe | 4 | 0 | - | - | - | - |
| **Total** |  | **452** | **32 (7.08%)** | **167** | **2 (1.20%)** | **89** | **1 (1.12%)** |
